# Asymmetric dimethylarginine and long-term adverse cardiovascular events in patients with type 2 diabetes: relation with the glycemic control

**DOI:** 10.1186/s12933-014-0156-1

**Published:** 2014-12-03

**Authors:** Chiao-Po Hsu, Pai-Feng Hsu, Ming-Yi Chung, Shing-Jong Lin, Tse-Min Lu

**Affiliations:** Division of Cardiovascular Surgery, Department of Surgery, Taipei Veterans General Hospital, Taipei, Taiwan; School of Medicine, National Yang-Ming University, Taipei, Taiwan; Division of Cardiology, Department of Internal Medicine, Taipei Veterans General Hospital, Taipei, Taiwan; Department of Life Sciences and Institute of Genome Sciences, National Yang-Ming University, Taipei, Taiwan; Department of Medical Research and Education, Taipei Veterans General Hospital, Taipei, Taiwan

**Keywords:** Asymmetric dimethylarginine, Diabetes, Hemoglobin A1c, Nitric oxide

## Abstract

**Background and aims:**

Elevated plasma asymmetric dimethylarginine (ADMA) levels have been observed in patients with insulin resistance and diabetes, and have been reported to predict adverse cardiovascular events in type 2 diabetic patients. However, the relationship between ADMA and glycemic control in patients with type 2 diabetes remained controversial.

**Methods and results:**

We evaluated 270 patients with type 2 diabetes and measured their plasma ADMA and hemoglobin A1c (HbA1c) levels by high performance liquid chromatography. The mean age was 67 ± 12 years. The mean plasma ADMA and HbA1c level were 0.46 ± 0.09 μmol/l and 7.8 ± 1.6%, respectively. There was no significant correlation between plasma ADMA level and HbA1c level (r = −0.09, p = 0.13). During the median follow-up period of 5.7 years (inter-quartile range: 5.0 − 7.3 years), major adverse cardiovascular event (MACE, including cardiovascular death, myocardial infarction and stroke) was observed in 55 patients (20.4%). Multivariate Cox regression analysis revealed that the ADMA tertile was an independent risk factor for MACE (ADMA tertile III versus ADMA tertile I: p = 0.026, HR: 2.31, 95% CI: 1.10 − 4.81). The prognosis predictive power of ADMA disappeared in patients with well glycemic control (HbA1c ≤6.5%), and the ADMA-HbA1c interaction p value was 0.01.

**Conclusions:**

In patients with type 2 diabetes, ADMA might be an independent risk factor for long-term adverse cardiovascular events. However, ADMA was not correlated with serum HbA1c level, and in diabetic patients with HbA1c ≤6.5%, elevated ADMA level was no longer associated with increased risk of long-term prognosis. Our findings suggested that the prognosis predictive value of ADMA in type 2 diabetes might be modified by the glycemic control.

## Introduction

Cardiovascular disease is the major cause of mortality and morbidity for individuals with type 2 diabetes [1]. Endothelial dysfunction is present from the onset of type 2 diabetes and strongly related to its cardiovascular outcomes [2], and itself may lead to the development of insulin resistance and diabetes [3,4]. Asymmetric dimethylarginine (ADMA) is characterized as a circulating endogenous inhibitor of nitric oxide (NO) synthase by competing with L-arginine as the substrate [5,6]. Moreover, ADMA may increase oxidative stress by uncoupling the electron transport between NO synthase and L-arginine, which can lead to decrease in the production and availability of endothelium-derived NO [7,8]. Therefore, derangement of the L-arginine- NO pathway and increase of oxidative stress by ADMA have been implicated as important contributing factors for the development of endothelial dysfunction. Elevated plasma ADMA levels have been observed in patients with insulin resistance and type 1/2 diabetes [9-11], and have been reported to be associated with micro/macrovascular diabetic complications [12-14]. Although several prospective studies have demonstrated that elevated plasma ADMA level may predict adverse cardiovascular events in type 1/2 diabetic patients [15-17], some other studies showed conflicting results [18,19]. Furthermore, the relationship between ADMA and hyperglycemic control in patients with type 2 diabetes remained controversial [20,21]. An acute reduction of plasma ADMA levels by insulin infusion was demonstrated in young patens with type 1 diabetes [22], but the same group reported a paradoxical inverse association between blood sugar control (glycosylated hemoglobin, HbA1c) and ADMA [21]. Hence, although ADMA appears to be a novel pathogenic factor of diabetes, these evidences suggest that the interactions among ADMA and type 2 diabetes are complex and far from being completely understood. In the present study we aimed to investigate the relation between plasma ADMA levels and glycemic control, and evaluate their associations with long-term clinical outcomes in patients with type 2 diabetes.

## Methods

### Study population

From July 2006 to June 2009, 270 patients with type 2 diabetes were recruited at Taipei Veterans General Hospital. Exclusion criteria included patients with severe liver disease and end-stage renal disease, active infectious disease, chronic or acute inflammatory disease, malignancy, and unstable hemodynamic status. Thorough medical histories of all participants were recorded. Type 2 diabetes mellitus was diagnosed according to the diagnostic criteria defined by the American Diabetes Association in 2003 [23] or when the individual was receiving oral hypoglycemic agents or insulin injection therapy at the time of recruitment. All of the diabetic patients have medication with oral hypoglycemic agents (n = 203, 75.2%, including metformin, n = 159, 58.9%, sulfonylurea, n = 130, 48.1%, thiazolidinedione, n = 18, 6.7%, acarbose, n = 32, 11.9%), and the remaining patients receiving insulin therapy only (n = 44, 16.3%), or insulin combined with oral hypoglycemic agents (n = 23, 8.5%), while a few received only dietary therapy only (n = 14, 5.2%**)**. Hypertension was diagnosed according to the Seventh Joint National Committee criteria [24] or if the patient was receiving anti-hypertensive drugs. Patients were considered to have significant coronary artery disease (CAD) in the presence of ≥50% stenosis in at least one major coronary artery by coronary angiography. All medications, cigarette smoking and beverages containing alcohol or caffeine were withdrawn for at least 12 hours. Fasting blood samples were collected in EDTA tubes for the measurements of L-arginine, dimethylarginines, HbA1c and for other biochemical analyses. All patients were prospectively followed by monthly office visit or by telephone contact and chart review for the occurrence of the first-ever major adverse cardiovascular events (MACE) which is defined as cardiovascular death, stroke and non-fatal myocardial infarction. Cardiovascular death was diagnosed as any death with definite cardiovascular cause or any death that was not clearly attributed to a non-cardiovascular cause. Myocardial infarction was defined as the presence of significant new Q waves in at least 2 electrocardiographic leads or of symptoms compatible with myocardial infarction associated with increase in creatine kinase-MB fraction ≥3 times the upper limit of the reference range. Stroke was diagnosed by a neurologist on the basis of with new neurological deficit or imaging study. The study protocol was approved by the Institutional Review Board at Taipei-Veterans General Hospital and all participants provided written informed consent.

### Laboratory measurements

Blood samples were centrifuged at 3000 rpm 4°C for 10 minutes immediately after collection and plasma was frozen at-80°C until analysis. Plasma L-arginine, ADMA and symmetric dimethylarginine (SDMA) concentrations were determined by high performance liquid chromatography as described previously [25]. The recovery rate for ADMA was >90% and the within-assay and between-assay variation coefficients were not more than 7% and 8%, respectively. HbA1c was determined by cation-exchange high performance liquid chromatography using an auto-analyzer (Model HLC-G8, Tosoh, Tokyo, Japan). The body mass index (BMI) was obtained from the ratio of weight (kg) to height squared (m^2^). The estimated glomerular filtration rate (eGFR) was calculated according to the simplified version of the Modification of Diet in Renal Disease Study prediction equation formula, modified by Ma et al. for Chinese patients (eGFR =175 × plasma creatinine^-1.234^ × age^-0.179^ × 0.79 [if female]) [26].

### Statistical analysis

Continuous data are presented as the mean ± standard deviation or with confidence interval (CI) of 95%. The study population was divided into two groups according to the HbA1c concentration > or ≤6.5% or grouped into tertiles according to the plasma levels of ADMA. All and differences between groups were compared with two-sample *t*-tests or one-way analysis of variance (ANOVA). Categorical data were compared by Chi-square test or Fisher’s exact test. Pearson’s correlation coefficients were calculated to examine possible correlations between continuous variables. Actuarial event-free survival curves were estimated using the Kaplan-Meier method and compared by log-rank test. The multivariate Cox regression analysis was performed to determine the association with the risk of MACE in all subjects, and the variables tested included age, gender, body mass index (BMI), hypertension, ADMA, L-arginine, HbA1c, and serum creatinine level. The plasma ADMA level was tested as a continuous or categorical variable. To investigate the possibility of a differential effect of diabetic control in more detail, the interaction is expressed as the product of the binary categorical variables (HbA1c ≤6.5% versus HbA1c >6.5%) and ADMA tertile. The hazards ratio (HR) and 95% confidence interval (CI) were calculated. A p value of less than 0.05 was considered statistically significant. The SPSS 17.0 (SPSS Inc., Chicago, Illinois) software package was used for statistical analysis.

## Results

### Baseline characteristics of the study population

The mean age of the 270 patients was 67 ± 12 years, and most of the patients were male (n = 213, 78.9%) and had concomitant CAD (n = 211, 78.1%). The mean plasma ADMA level, SDMA level and L-arginine level were 0.46 ± 0.09 μmol/l, 0.76 ± 0.42μmol/l and 87.5 ± 29.4 μmol/l, respectively, and the mean HbA1c level was 7.8 ± 1.6%. Significant correlations were observed between plasma ADMA level and age (r = 0.21, p < 0.01), creatinine (r = 0.34, p < 0.01), SDMA (r = 0.44, p < 0.01), L-arginine (r = 0.22, p < 0.01) and eGFR (r = −0.28, p < 0.01), respectively. In contrast, there was no significant correlation between plasma ADMA level and HbA1c concentration (r = −0.09, p = 0.13). Notably, the plasma ADMA levels of patients receiving metformin were significantly lower than those without (0.45 ± 0.08 μmol/l vs 0.48 ± 0.09 μmol/l, p < 0.01). Furthermore, there was a trend toward higher plasma ADMA level in patients receiving insulin therapy (p = 0.07). The baseline characteristics of patients grouped according to ADMA tertiles are shown in Table 1. There were no significant differences in baseline clinical parameters among the three groups, with the exception that the patients in higher ADMA tertiles were older and had worse renal function (Table1).

**Table 1:** **Clinical characteristics of study population grouped by ADMA tertile**

	**Tertile I**	**Tertile II**	**Tertile III**	
	**n = 90**	**n = 90**	**n = 90**	**P value**
Age (years)	63 ± 12	68 ± 11	70 ± 11	< 0.01
Gender (men,%)	73 (81)	67 (74)	73 (81)	0.51
BMI (Kg/m^2^)	27 ± 3	27 ± 5	27 ± 4	0.45
Hypertension (%)	78 (87)	71 (79)	76 (84)	0.39
Smoking (%)	22 (24)	16 (18)	15 (17)	0.40
Hypercholesterolemia (%)	40 (44)	40 (44)	40 (44)	1.00
CAD (%)	66 (73)	69 (77)	76 (84)	0.18
Cholesterol (mg/dl)				
Total	166 ± 31	163 ± 35	157 ± 31	0.19
HDL-Cholesterol	40 ± 11	40 ± 8	41 ± 14	0.13
LDL-Cholesterol	100 ± 27	101 ± 31	93 ± 25	0.82
Triglyceride (mg/dl)	167 ± 92	155 ± 96	166 ± 102	0.68
Creatinine (mg/dl)	1.1 ± 0.4	1.2 ± 0.4	1.8 ± 1.7	<0.01
eGFR (ml/min per 1.73 m^2^)	79 ± 28	70 ± 30	57 ± 33	<0.01
Fasting blood sugar (mg/dl)	137 ± 54	139 ± 51	136 ± 42	0.90
ADMA (μmol/l)	0.37 ± 0.04	0.45 ± 0.02	0.56 ± 0.06	<0.01
SDMA (μmol/l)	0.60 ± 0.18	0.69 ± 0.25	0.99 ± 0.60	<0.01
L-arginine (μmol/l)	80 ± 26	89 ± 28	94 ± 32	0.01
L-arginine/ADMA	216 ± 72	197 ± 64	169 ± 57	<0.01
HbA1c	7.7 ± 1.7	8.0 ± 1.6	7.8 ± 1.4	0.50
*Hypoglycemic treatments*				
Sulfaurea (%)	42 (47)	47 (52)	49 (46)	0.65
Metformin (%)	63 (70)	48 (53)	48 (53)	0.04
TZD (%)	6 (7)	6 (7)	6 (7)	1.0
Acarbose (%)	12 (13)	14 (16)	3 (7)	0.16
Insulin (%)	16 (18)	23 (26)	28 (31)	0.11
Insulin + OHA (%)	6 (7)	11 (12)	6 (7)	0.35

BMI: body mass index; CAD: coronary artery disease; eGFR: estimated glomerular filtration rate; OHA: oral hypoglycemic agent; TZD: thiazolidinedie.

### Long-term outcome and dimethylarginines

**Figure 1:**
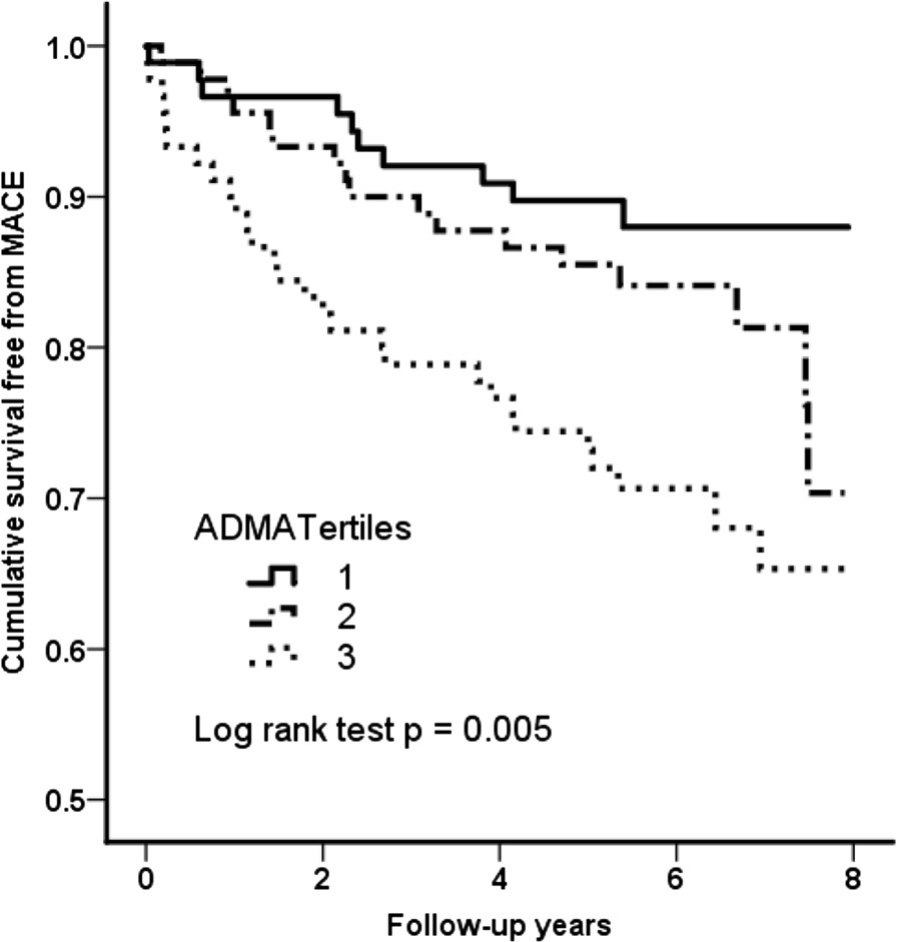
**Kaplan-Meier survival analyses for major adverse cardiovascular event during follow-up according to the plasma ADMA tertiles.** P values by log-rank test are shown.

All patients were followed up completely for a median period of 5.7 years (inter-quartile range: 5.0 − 7.3 years). During the follow-up period, MACE was observed in 55 patients (20.4%), which included 36 cardiovascular death (13.3%), 16 non-fatal MI (6.0%) and 7 stroke (2.6%). The plasma ADMA levels in patients who developed MACE were significantly higher than in those who did not (0.49 ± 0.09 μmol/l versus 0.45 ± 0.09 μmol/l, p = 0.006). By Kaplan-Meier analysis, event-free survival from MACE was significantly associated with ADMA tertiles (p < 0.005), with outcome being the worst in those patients with the highest plasma ADMA levels (Figure 1).

In multivariate Cox regression analysis adjusted for age, sex, BMI, HbA1c, L-arginine, hypertension and serum creatinine level, patients in the highest ADMA tertile was independently associated with a 2.31-fold increased risk for MACE when compared with patients with the lowest ADMA tertile (p = 0.026, HR: 2.31, 95% CI: 1.10 − 4.81). When considering the plasma ADMA level as a continuous variable, the plasma ADMA level remained a significant independent predictor for the occurrence of MACE, and the relative risk of MACE increased by 30% when plasma ADMA level increased by 1 SD of value (p = 0.04, HR: 1.30, 95% CI: 1.01 − 1.68).

The plasma SDMA level was significantly associated with long-term MACE in uni-variate analysis (p < 0.01, HR: 1.30, 95% CI: 1.14 − 1.48). However, when we put both ADMA and SDMA into the multivariate Cox regression analysis, the significance of SDMA disappeared (p = 0.21).

### The relationship between ADMA and the control of DM

To investigate the relationship of diabetic control and ADMA, we divided all patients into those with plasma HbA1c level ≤6.5% (n = 50) and >6.5% (n = 220). The baseline characteristics of both groups are shown in Table 2. The plasma ADMA level of both groups were similar, and there were no significant differences in baseline clinical parameters between the two groups, with the exception that the patients with better diabetic control received less sulfaurea and insulin treatment, and had higher HDL and lower triglyceride levels (Table 2).

**Table 2:** **Clinical characteristics of study population grouped by glycemic control**

	**HbA1c ≤6.5%**	**HbA1c >6.5%**	
	**n = 50**	**n = 220**	**P value**
Age (years)	68 ± 12	67 ± 12	0.66
Gender (men,%)	40 (80)	173 (79)	0.85
BMI (Kg/m^2^)	27 ± 4	27 ± 4	0.60
Hypertension (%)	43 (86)	182 (83)	0.67
Smoking (%)	7 (14)	46 (21)	0.33
Hypercholesterolemia (%)	19 (38)	101 (46)	0.35
CAD (%)	36 (72)	175 (80)	0.26
Cholesterol (mg/dl)			
Total	159 ± 33	164 ± 33	0.39
HDL-Cholesterol	44 ± 16	40 ± 9	0.04
LDL-Cholesterol	95 ± 27	99 ± 29	0.40
Triglyceride (mg/dl)	138 ± 68	170 ± 105	0.04
Creatinine (mg/dl)	1.4 ± 1.0	1.4 ± 1.1	0.96
eGFR (ml/min per 1.73 m^2^)	69 ± 29	69 ± 32	0.88
Fasting blood sugar (mg/dl)	113 ± 25	143 ± 51	<0.01
ADMA (μmol/l)	0.47 ± 0.10	0.46 ± 0.08	0.27
SDMA (μmol/l)	0.70 ± 0.37	0.81 ± 0.60	0.33
L-arginine (μmol/l)	87 ± 30	88 ± 29	0.83
L-arginine/ADMA	188 ± 69	195 ± 67	0.52
HbA1c	6.1 ± 0.3	8.2 ± 1.5	<0.01
*Hypoglycemic treatments*			
Sulfaurea (%)	17 (34)	113 (51)	0.03
Metformin (%)	29 (58)	130 (59)	1.00
TZD (%)	1 (2)	17 (8)	0.21
Acarbose (%)	2 (4)	30 (14)	0.09
Insulin (%)	5 (10)	62 (28)	<0.01
Insulin + OHA (%)	1 (2)	22 (10)	0.09

BMI: body mass index; CAD: coronary artery disease; eGFR: estimated glomerular filtration rate; OHA: oral hypoglycemic agent; TZD: thiazolidinedie.

**Figure 2:**
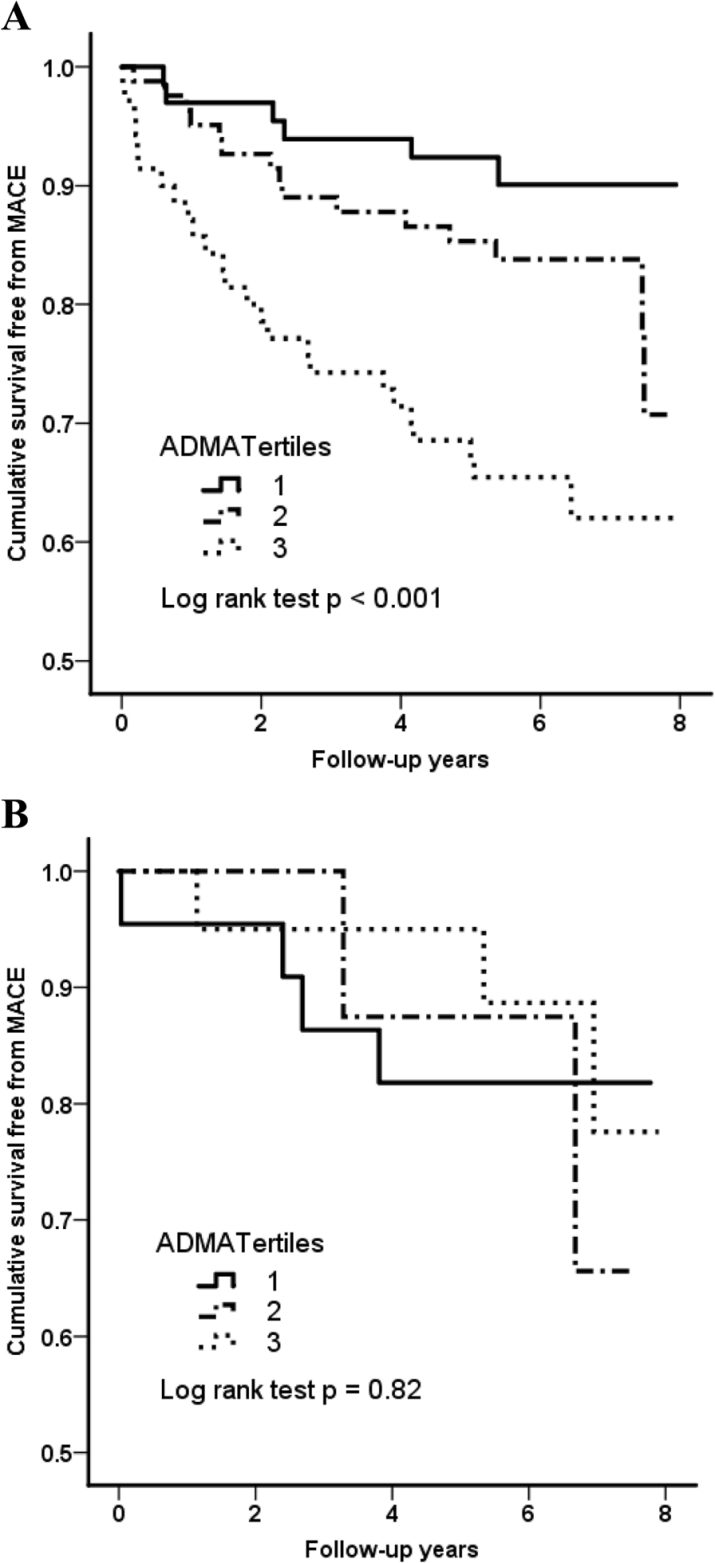
**Kaplan-Meier survival analyses for major adverse cardiovascular event during follow-up according to the plasma ADMA tertiles in subgroup with HbA1c >6.5% (A) and subgroup with HbA1c** ≤**6.5% (B).** P values by log-rank test are shown.

Table 3 showed the MACE rate stratified according to the ADMA tertiles and HbA1c level. By Kaplan-Meier analysis, the higher plasma ADMA tertiles were still associated with increased risk of MACE in subgroup with serum HbA1c >6.5%, but not in subgroup with HbA1c ≤6.5% (Figure 2A, B). In multivariate analysis, ADMA remained an independent significant predictor for MACE in subgroup with HbA1c >6.5% (tertile III versus tertile I: p = 0.02, HR: 3.0, 95% CI: 1.2 − 7.7; ADMA as a continuous variable: p = 0.04; HR per increase of 1 SD: 1.38, 95% CI: 1.0 − 1.9), but not in patients with HbA1c ≤6.5% (Table 4). There was a significant interaction between the predictive power of ADMA tertiles for the MACE risk and diabetic control (interaction p = 0.01). In contrast, glycemic control was not associated with long-term cardiovascular outcomes (HbA1c ≤6.5% versus HbA1c >6.5%: p = 0.57 by log-rank test).

**Table 3:** **Major adverse cardiovascular events during clinical follow-up – stratified according to ADMA tertiles and glycemic control**

	**ADMA Tertiles**			
	**Tertile I**	**Tertile II**	**Tertile III**	p value*
HbA1c ≤6.5% (n = 50)	4 (18.2%)	2 (25.0%)	3 (15.0%)	0.82*
HbA1c >6.5% (n = 220)	6 (8.8%)	15 (18.3%)	25 (35.7%)	<0.001*

MACE: major adverse cardiovascular events, including cardiovascular death, non-fatal myocardial infarction and stroke; *p value by log-rank test.

**Table 4:** **Univariate and multivariate Cox regression analyses for major adverse cardiovascular events**

	***Univariate analysis***		***Multivariate analysis***	
	**HR (95% CI)**	**P**	**HR (95% CI)**	**P**
Age (years)	1.05 (1.02 – 1.08)	<0.01	1.05 (1.02 – 1.07)	<0.01
Gender	1.89 (0.85 – 4.17)	0.12	-	-
BMI	0.96 (0.89 –1.02)	0.20	-	-
Hypertension	0.94 (0.47 – 1.86)	0.80	-	-
Creatinine	1.10 (0.95 – 1.28)	0.20	-	-
HbA1c	1.05 (0.89 – 1 .23)	0.58	-	-
Hypercholesterolemia	0.69 (0.40 – 1.12)	0.18	-	-
Smoking	0.62 (0.28 – 1.37)	0.24	-	-
SDMA	1.30 (1.14 − 1.48)	<0.01	-	-
L-arginine	0.99 (0.98 – 1.00)	0.12	-	-
ADMA				
Tertile II versus tertile I	1.64 (0.75 – 3.60)	0.21	1.33 (0.60 – 2.93)	0.48
Tertile III versus tertile I	2.99 (1.45 – 6.16)	<0.01	2.31 (1.11 – 4.81)	0.026
**HbA1c ≤6.5%**				
ADMA tertile III vs I			0.62 (0.14 – 2.83)	0.54
**HbA1c >6.5%**				
ADMA tertile III vs I			3.33 (1.35 – 8.26)	<0.01

## Discussion

The results of this study showed that plasma ADMA level was a significant independent risk factor for long-term adverse cardiovascular events in patients with type 2 diabetes, especially in patients with serum HbA1C >6.5%. In contrast, ADMA was not correlated with serum HbA1c level. Furthermore, in type 2 diabetic patients with well glycemic control, higher plasma ADMA level was not associated with higher risk of long-term MACE. Our findings suggested that the predictive power of ADMA in type 2 diabetes might be modified by the glycemic control.

It is well recognized that type 2 diabetes and its metabolic derangements such as hyperinsulinemia, hyperglycemia, dyslipidemia, and increased oxidative stress are associated with NO-mediated endothelial dysfunction [2]. In contrast, endothelial dysfunction may lead to attenuated glucose uptake in insulin-sensitive tissues, hyperglycemia, and ultimately to the development of insulin resistance and type 2 diabetes [3,4]. Furthermore, impaired NO bioavailability plays a pivotal role in the regulation of glucose-stressed endothelial progenitor cell dysfunction in type 2 diabetes, and anti-oxidant treatment with superoxide dismutase may restore their function [27]. ADMA, an endogenous competitive inhibitor of NO synthase, is known to impair NO bioavailability and endothelial function [5-8]. Over-expression of dimethylaminohydrolase (*DDAH,* the enzyme responsible for the elimination of ADMA in human) may lead to reduced plasma ADMA level and enhance insulin sensitivity [28]. In human studies, insulin resistance is related to plasma ADMA concentration and treatment with rosiglitazone and weight reduction enhance insulin sensitivity and significantly lower plasma ADMA level [9,29]. Plasma ADMA level has been reported to be correlated to treadmill stress test derived outcome parameters [30] and asymptomatic carotid atherosclerosis [31]. In pre-diabetic subjects, elevated ADMA was strongly associated with increased arterial stiffness, which is a well-established predictor of cardiovascular outcomes [32]. Furthermore, plasma ADMA level might predict the future deterioration of glucose tolerance during 4.5-year follow-up independent of baseline insulin resistance [33]. Several studies have showed that the ADMA concentrations might be increased in patients with diabetes [9-11]. Elevated ADMA level was reported to be associated with the development and progression of diabetic complications [12-14] as well as worse long-term cardiovascular prognosis in diabetic patients [15-17]. Furthermore, ADMA was associated with the coronary atherosclerosis and its extent and severity both in non-diabetic and diabetic patients [34,35]. However, in a large-scale study of 3238 individuals scheduled for coronary angiography, the plasma ADMA levels of patients with type 2 diabetes were only marginally higher than those of the patients without diabetes (0.83 μmol/l *versus* 0.82 μmol/l, p = 0.032) [36]. In another large community-based population study enrolling 3320 Framinghan Offspring Study participants, the mean plasma ADMA level was similar between non-diabetic and diabetic subjects (0.546 μmol/l *versus* 0.553 μmol/l, p = NS). Furthermore, though in whole population plasma ADMA level was associated with all-cause mortality during a follow-up period of 10.9 years, it is surprising to find that this association was not evident in subgroup with diabetes [19]. A recent study involving 783 older type 2 diabetic patients showed that plasma ADMA level was not associated with glycemic control and incident cardiovascular disease during the follow-up period of 4 years [37]. Interestingly, Sibal et al. reported recently that the plasma ADMA levels in patients with early type 1 diabetes without macrovascular disease or macroalbuminuria were even significantly lower compared to healthy controls. In addition, the plasma ADMA levels were not associated with impaired flow-mediated dilatation of brachial arteries in these diabetic patients [38]. Our study showed that plasma ADMA level was a significant independent risk factor for long-term adverse cardiovascular events only in type 2 diabetic patients with poor glycemic control (namely in patients with serum HbA1c >6.5%). As the mean values of HbA1c of diabetic patients in most of the previous studies showing that ADMA was a prognostic marker in diabetes were >7.0%, the results of our study might partially explain the conflicting results of previous studies, suggesting that the involvement of ADMA in the pathogenesis of diabetes and its predicting value of long-term prognosis might be evident only in patients with advanced/complicated diabetes as well as without intensive glycemic control. It has been speculated that inhibition of uncoupled endothelial NO synthase by ADMA with resulting paradoxical reduction of oxidative stress might be a possible explanation for the paradoxical association of ADMA with cardiovascular events in patients with uncomplicated diabetes [39], and maybe also in patients with intensive glycemic control. On the other hand, intensive glycemic control was not associated with less long-term adverse events in our study and in other large trial, and even associated with higher cardiovascular mortality rate in the ACCORD study [40-42]. Hypoglycemia associated with intensive glycemic control and other unknown mechanisms might attenuate the prognosis predictive power of ADMA. Nevertheless, the relation between ADMA and diabetes/glycemic control seems to be more complex and remains to be elucidated.

The relationship between ADMA and diabetic control is not clear. Some studies showed that plasma concentrations were inversely correlated with HbA1c in patients with type 1/2 diabetes [18,20]. In contrast, an acute reduction of plasma ADMA levels by insulin was demonstrated in 15 young patients with type1 diabetes [22]. Confusingly, Devangelio et al. showed that plasma ADMA level positively correlated with HbA1c and oxidative stress [20]. Another small study in type 2 diabetes showed higher ADMA concentrations in patients with poor glycemic control (HbA1c >6.5%) [43]. In addition, intensive treatment of hyperglycemia was associated with improvement of endothelial dysfunction and decrease of plasma ADMA levels [44]. On the other hand, Krzyzanowska et al. reported that in 125 patients with type 2 diabetes the plasma ADMA concentrations was not correlated with HbA1c [15]. In other two larger cross-sectional study involving more than 500 type 2 diabetes patients and 783 older type 2 diabetes patients respectively, both showed a lack of significant correlation between ADMA and HbA1c [37,45]. It is speculated that chronic hyperglycemia may increase proteolysis and plasma ADMA levels concomitantly with reduced ADMA metabolism by DDAH. To the contrary, hyperfiltration and increased renal clearance driven by hyperglycemia has been suggested as the major mechanism responsible for increased excretion of ADMA and possible inverse correlation with HbA1c [18]. Although a significant negative correlation between ADMA and eGFR was found, the plasma ADMA concentration was insignificantly correlated with HbA1c in our study. Different study design, different ethnicity and clinical characteristics of the study population may lead to these conflicting results, and the relation between ADMA and glycemic control need further studies.

### Limitations

Several limitations of this study need to be addressed. First, this study was a single-center observational study, and most of the patients had CAD and many concomitant atherosclerotic risk factors in addition to diabetes, which might have a potential impact on the long-term outcomes. Second, our sample size was small, especially the group with intensive glycemic control. Confirmation of our findings in a cohort involving more patients with well glycemic control is needed. Third, we evaluate the relation of baseline ADMA and HbA1c levels with long-term prognosis, but the long-term glycemic control is uncertain. Finally, as previous studies have shown that metformin, thiazolidinedies and insulin may affect the plasma ADMA levels [9,18], their use for control of diabetes might result in bias in this study. Although the use of these hypoglycemic agents were largely comparable in the ADMA tertiles subgroups and subgroup of HbA1c ≤6.5% and >6.5%, with the exceptions that more use of metformin in patients in the lowest ADMA tertiles and more use of insulin in the HbA1c >6.5% subgroup, we cannot exclude completely the effects of these medication on the results of our study even after the adjustment of multivariate analysis.

## Conclusions

In patients with type 2 diabetes, ADMA might be an independent risk factor for long-term adverse cardiovascular events. However, ADMA was not correlated with serum HbA1c level, and in diabetic patients with well glycemic control, namely HbA1c ≤6.5%, elevated ADMA level was no longer associated with increased risk of long-term prognosis. Our findings suggested that the prognosis predictive value of ADMA in type 2 diabetes might be modified by the glycemic control.

## Abbreviations

ADMA: Asymmetric dimethylarginine
BMI: Body mass index
CAD: Coronary artery disease
DDAH: Dimethylarginine dimethylaminohydrolase
eGFR: estimated glomerular filtration rate
HbA1c: Glycosylated hemoglobin
MACE: Major adverse cardiovascular events
NO: Nitric oxide
SDMA: Symmetric dimethylarginine

## References

[1] Haffner SM, Lehto S, Ronnemaa T, Pyorala K, Laakso M (1998). Mortality from coronary heart disease in subjects with type 2 diabetes and in nondiabetic subjects with and without prior myocardial infarction. N Engl J Med.

[2] Schalkwijk CG, Stehouwer CD (2005). Vascular complications in diabetes mellitus: the role of endothelial dysfunction. Clin Sci.

[3] Steinberg HO, Brechtel G, Johnson A, Fineberg N, Baron AD (1994). Insulin-mediated skeletal muscle vasodilation is nitric oxide dependent. A novel action of insulin to increase nitric oxide release. J Clin Invest.

[4] Vincent MA, Clerk LH, Lindner JR, Klinbanov AL, Clark MG, Ratiggan S, Barrett EJ (2004). Microvascular recruitment is an early insulin effect that regulates skeletal muscle glucose uptake in vivo. Diabetes.

[5] Kakimoto Y, Akazawa S (1970). Isolation and identification of N^G^, N^G-^ and N^G^, N^’G^ dimethyl-arginine, N^e^-mono-, di-, and trimethyllysine, glucosylgalactosyl- and galactosyl-delta-hydroxylysine from human urine. J Biol Chem.

[6] Vallance P, Leone A, Calver A, Collier J, Moncada S (1992). Accumulation of an endogenous inhibitor of nitric oxide synthesis in chronic renal failure. Lancet.

[7] Leiper J, Vallance P (1999). Biological significance of endogenous methylarginines that inhibit nitric oxide synthases. Cardiovasc Res.

[8] Böger RH, Bode-Böger SM, Tsao PS, Lin PS, Chan JR, Cooke JP (2000). An endogenous inhibitor of nitric oxide synthase regulates endothelial adhesiveness for monocytes. J Am Coll Cardiol.

[9] Stühlinger MC, Abbasi F, Chu JW, Lamendola C, McLaughlin TL, Cooke JP, Reaven GM, Tsao PS (2002). Relationship between insulin resistance and an endogenous nitric oxide synthase inhibitor. JAMA.

[10] Abbasi F, Asagmi T, Cooke JP, Lamendola C, McLaughlin T, Reaven GM, Stühlinger M, Tsao PS (2001). Plasma concentrations of asymmetric dimethylarginine are increased in patients with type 2 diabetes mellitus. Am J Cardiol.

[11] Altinova AE, Arslan M, Sepici-Dincel A, Akturk M, Altan N, Toruner FB (2007). Uncomplicated type 1 diabetes is associated with increased asymmetric dimethylarginine concentrations. J Clin Endocrinol Metab.

[12] Krzyzanowska K, Mittermayer F, Krugluger W, Schnack C, Hofer M, Wolzt M, Schernthaner G (2006). Asymmetric dimethylarginine is associated with macrovascular disease and total homocysteine in patients with type 2 diabetes. Atherosclerosis.

[13] Abhary S, Kasmeridis N, Burdon KP, Kuot A, Whiting MJ, Yew WP, Petrovsky N, Craig JE (2009). Diabetic retinopathy is associated with elevated serum asymmetric and symmetric dimethylarginines. Diabetes Care.

[14] Hanai K, Babazono T, Nyumura I, Toya K, Tanaka N, Tanaka M, Ishii A, Iwamoto Y (2009). Asymmetric dimethylarginine is closely associated with the development and progression of nephropathy in patients with type 2 diabetes. Nephro Dial Transplant.

[15] Krzyzanowska K, Wolzt M, Mittermayer F, Schernthaner G (2007). Asymmetric dimethylarginine predicts cardiovascular events in patients with type 2 diabetes. Diabetes Care.

[16] Lajer M, Teerlink T, Tarnow L, Parving H, Jorsal A, Rossing P (2008). Plasma concentration of asymmetric dimethylarginine (ADMA) predicts cardiovascular morbidity and mortality in type 1 diabetic patients with diabetic nephropathy. Diabetes Care.

[17] Cavusoglu E, Ruwende C, Chopra V, Poludasu S, Yanamadala S, Frishman WH, Eng C, Pinsky DJ, Marmur JD (2010). Relation of baseline ADMA levels to cardiovascular morbidity and mortality at two years in men with diabetes referred for coronary angiography. Atherosclerosis.

[18] Päivä H, Lehtimäki, Laakso J, Ruokonen I, Rantaiho V, Wirta O, Pasternack A, Laaksonen R (2003). Plasma concentrations of asymmetric dimethylarginine in type 2 diabetes associated with glycemic control and glomerular filtration rate but not with risk factors of vasculopathy. Metabolism.

[19] Böger RH, Sullivan LM, Schwedhelm E, Wang TJ, Maas R, Benjamin EJ, Schulze F, Xanthakis V, Benndorf RA, Vasan RS (2009). Plasma asymmetric dimethylarginine and incidence of cardiovascular disease and death in the community. Circulation.

[20] Devangelio E, Santilli F, Formoso G, Ferroni Bucciarelli L, Michetti N, Clissa C, Ciabattoni G, Consoli A, Davi G (2007). Soluble RAGE in type 2 Diabetes: association with oxidative stress. Free Radial Biol Med.

[21] Marcovecchio ML, Widmer B, Turner C, Dunger B, Dalton RN (2011). Asymmetric dimethylarginine in young people with type 1 diabetes: a paradoxical association with HbA1c. Diabetic Med.

[22] Marcovecchio ML, Widmer B, Dunger B, Dalton RN (2008). Effect of acute variations of insulin and glucose on plasma concentrations of asymmetric dimethylarginine in young people with type 1 diabetes. Clin Sci.

[23] The Expert Committee on the Diagnosis and Classification of Diabetes Mellitus (2003). Follow-up report on the diagnosis of diabetes mellitus. Diabetes Care.

[24] Chobanian AV, Bakris GL, Black HR, Cushman WC, Green LA, Izzo JL, Jones DW, Materson BJ, Oparil S, Wright JT, Roccella EJ, and the National High Blood Pressure Education Program Coordinating Committee (2003). The seventh report of the Joint National Committee on prevention, detection, evaluation, and treatment of high blood pressure. JAMA.

[25] Lu TM, Ding YA, Lin SJ, Lee WS, Tai HC (2003). Plasma levels of asymmetrical dimethylarginine and adverse cardiovascular events after percutaneous coronary intervention. Eur Heart J.

[26] Ma YC, Zuo L, Chen JH, Luo Q, Yu XQ, Li Y, Xu JS, Huang SM, Wang LN, Huang W, Wang M, Xu GB, Wang HY (2006). Modified glomerular filtration rate estimating equation for Chinese patients with chronic kidney disease. J Am Soc Nephrol.

[27] Hamed S, Brenner B, Aharon A, Daoud D, Roguin A (2009). Nitric oxide and superoxide dismutase modulate endothelial progenitor cell function in type 2 diabetes mellitus. Cardiovasc Diabetol.

[28] Sydow K, Mondon CE, Schrader J, Konishi H, Cooke JP (2008). Dimethylarginine dimethylaminohydrolase overexpression enhances insulin sensitivity. Arterioscler Thromb Vasc Biol.

[29] Mclaughlin T, Stühlinger M, Lamendola C, Abbasi F, Bilek J, Reaven GM, Tsao PS (2006). Plasma asymmetric dimethylarginine concentrations are elevated in obese insulin-resistant women and fall with weight loss. J CLin Endocrine Metab.

[30] Deftereos S, Bouras G, Tsounis D, Papadimitriou C, Hatzis G, Raisakis K, Panagopoulou V, Kaoukis A, Ioannidis A, Deftereos G, Kossyvakis C, Manolis AS, Alexopoulos D, Stefanadis C, Cleman MW, Giannopoulos G (2014). Association of asymmetric dimethylarginine levels with treadmill-stress-test-derived prognosticators. Clin Biochem.

[31] Riccioni G, Scotti L, D'Orazio N, Gallina S, Speziale G, Speranza L, Bucciarelli T (2014). ADMA/SDMA in elderly subjects with asymptomatic carotid atherosclerosis: values and site-specific association. Int J Mol Sci.

[32] Protopsaltis I, Foussas S, Angelidi A, Gritzapis A, Sergentanis T, Matsagos S, Tzirogiannis K, Panoutsopoulos GI, Dimitriadis G, Raptis S, Melidonis A (2012). Impact of ADMA, endothelial progenitor cells and traditional cardiovascular risk factors on pulse wave velocity among prediabetic individuals. Cardiovasc Diabetol.

[33] Surdacki A, Kruszelnicka O, Rakowski T, Jaźwińska-Kozuba A, Dubiel JS (2013). Asymmetric dimethylarginine predicts decline of glucose tolerance in men with stable coronary artery: a 4.5-year follow-up study. Cardiovasc Diabetol.

[34] Kruszelnicka O, Surdacki A, Golay A (2013). Differential associations of angiographic extent and severity of coronary artery disease with asymmetric dimethylarginine but not insulin resistance in non-diabetic men with stable angina: a cross-sectional study. Cardiovasc Diabetol.

[35] Jawalekar SL, Karnik A, Bhutey A: **Risk of cardiovascular disease in diabetes mellitus and serum concentration of asymmetrical dimethylarginine.** *Biochem Res Int* 2013, 189430. 10.1155/2013/189430 PMC3804277 24187621

[36] Meinitzer A, Seelhorst U, Wellnitz B, Halwachs-Baumann G, Boehm B, Winkelmann BR, März W (2007). Asymmetrical dimethylarginine independently predicts total cardiovascular mortality in individuals with angiographic coronary artery disease (The Ludwigshafen risk and cardiovascular health study). Clin Chem.

[37] Anderssohn M, McLachlan S, Lüneburg N, Robertson C, Schwedhelm E, Williamson RM, Strachan MW, Ajjan R, Grant PJ, Böger RH, Price JF (2014). Genetic and environmental determinants of dimethylarginines and association with cardiovascular disease in patients with type 2 diabetes. Diabetes Care.

[38] Sibal L, Agarwal SC, Schwedhelm E, Lüneburg N, Böger RH, Home PD (2009). A study of endothelial function and circulating asymmetric dimethylarginine levels in people with type I diabetes without macrovascular disease or macroalbuminuria. Cardiovasc Diabetol.

[39] Anderssohn M, Schwehelm E, Lüneburg N, Vasan SR, Böger RH (2010). Asymmetric dimethylarginine as a mediator of vascular dysfunction and a marker of cardiovascular disease and mortality: an intriguing interaction with diabetes mellitus. Diabetes Vasc Dis Res.

[40] Patel A, MacMahon S, Chalmers J, Neal B, Billot L, Woodwars M, Marre M, Cooper M, Glasziou P, Grobbee D, Hamet P, Harrap S, Heller S, Liu L, Mancia G, Mogensen CE, Pan C, Poulter N, Rodger A, Williams B, Bompoint S, De Galan BE, Joshi R, Travert F, ADVANCE Collaborative Group (2008). Intensive blood glucose control and vascular outcomes in patients with type 2 diabetes. N Engl J Med.

[41] Duckworth W, Abraira C, Mortiz T, Reda D, Emanuele N, Reaven PD, Zieve FJ, Marks J, Davis SN, Hayward R, Warren SR, Goldman S, McCarren M, Vitek ME, Henderson WG, Huang GD (2009). Glucose control and vascular complications in veterans with type 2 diabetes. N Engl J Med.

[42] Gerstein HC, Miller ME, RP B t, Goff DC, Bigger JT, Buse JB, Cushman WC, Genuth S, Imail-Beigi F, Grimm RH, Probstfield JL, Simons-Morton DG, Friedewald WT (2008). Effects of intensive glucose lowering in type 2 diabetes. N Engl J Med.

[43] Can A, Bekpinar S, Gurdol F, Tutuncu Y, Unlucerci Y, Dinccag N (2011). Dimethylarginines in patients with type 2 diabetes mellitus: relation with the glycaemic control. Diabetes Res Clin Pract.

[44] Yasuda S, Miyazaki S, Kanda M, Goto Y, Suzuki M, Harano Y, Nonogi H (2006). Intensive treatment of risk factors in patients with type 2 diabetes mellitus is associated with improvement of endothelial function coupled with a reduction in the levels of plasma asymmetric dimethylarginine and endogenous inhibitor of nitric oxide synthase. Eur Heart J.

[45] Kanazawa I, Yano S, Notsu Y, Yamaguchi T, Nabika T, Sugimoto T (2011). Asymmetric dimethylarginine as a risk factor for cardiovascular disease in Japanese patients with type 2 diabetes mellitus. Clin Endocrinology.

